# Cord-Blood Lipidome in Progression to Islet Autoimmunity and Type 1 Diabetes

**DOI:** 10.3390/biom9010033

**Published:** 2019-01-21

**Authors:** Santosh Lamichhane, Linda Ahonen, Thomas Sparholt Dyrlund, Alex M. Dickens, Heli Siljander, Heikki Hyöty, Jorma Ilonen, Jorma Toppari, Riitta Veijola, Tuulia Hyötyläinen, Mikael Knip, Matej Oresic

**Affiliations:** 1 Turku Centre for Biotechnology, University of Turku and Åbo Akademi University, 20520 Turku, Finland; santosh.lamichhane@utu.fi (S.L.); alex.dickens@utu.fi (A.M.D.); 2 Steno Diabetes Center Copenhagen, 2820 Gentofte, Denmark; la@biosyntia.com (L.A.); thomas@dyrlund.dk (T.S.D.); 3 Children’s Hospital, University of Helsinki, Helsinki University Hospital and Research Program Unit, Diabetes and Obesity, University of Helsinki, 00290 Helsinki, Finland; heli.siljander@helsinki.fi; 4 Faculty of Medicine and Life Sciences, University of Tampere, 33014 Tampere, Finland; heikki.hyoty@uta.fi; 5 Fimlab Laboratories, Pirkanmaa Hospital District, 33014 Tampere, Finland; 6 Immunogenetics Laboratory, Institute of Biomedicine, University of Turku, 20520 Turku, Finland; jsilonen@utu.fi; 7 Clinical Microbiology, Turku University Hospital, 20014 Turku, Finland; 8 Institute of Biomedicine, Centre for Integrative Physiology and Pharmacology, University of Turku, 20014 Turku, Finland; jortop@utu.fi; 9 Department of Pediatrics, Turku University Hospital, 20521 Turku, Finland; 10 Department of Pediatrics, PEDEGO Research Unit, Medical Research Centre, University of Oulu, 90014 Oulu, Finland; riitta.veijola@oulu.fi; 11 Department of Children and Adolescents, Oulu University Hospital, 90220 Oulu, Finland; 12 Department of Women’s and Children’s Health, Karolinska Institutet, 17177 Stockholm, Sweden; 13 School of Science and Technology, Örebro University, 70281 Örebro, Sweden; Tuulia.Hyotylainen@oru.se; 14 Tampere Center for Child Health Research, Tampere University Hospital, 33520 Tampere, Finland; 15 Folkhälsan Research Center, 00290 Helsinki, Finland; 16 School of Medical Sciences, Örebro University, 702 81 Örebro, Sweden

**Keywords:** type 1 diabetes, cord blood, lipidomics, metabolomics, autoimmunity

## Abstract

Previous studies suggest that children who progress to type 1 diabetes (T1D) later in life already have an altered serum lipid molecular profile at birth. Here, we compared cord blood lipidome across the three study groups: children who progressed to T1D (PT1D; *n* = 30), children who developed at least one islet autoantibody but did not progress to T1D during the follow-up (P1Ab; *n* = 33), and their age-matched controls (CTR; *n* = 38). We found that phospholipids, specifically sphingomyelins, were lower in T1D progressors when compared to P1Ab and the CTR. Cholesterol esters remained higher in PT1D when compared to other groups. A signature comprising five lipids was predictive of the risk of progression to T1D, with an area under the receiver operating characteristic curve (AUROC) of 0.83. Our findings provide further evidence that the lipidomic profiles of newborn infants who progress to T1D later in life are different from lipidomic profiles in P1Ab and CTR.

## 1. Introduction

Type 1 diabetes (T1D) is an autoimmune disease, characterized by destruction of insulin-producing pancreatic islet β-cells that results in lifelong dependency on exogenous insulin [1]. The incidence of T1D is steadily increasing in children younger than 15 years of age and is projected to double in children under the age of five years by 2020 [2]. No effective T1D prevention strategies have so far been identified. Early detection of T1D risk has, consequently, become an important area of research, which may also inform about potential disease prevention strategies.

The presence of predisposing human leukocyte antigen (HLA) alleles in the human genome is a major determinant of T1D susceptibility, but less than 10% of individuals with high-risk HLA alleles progress to the disease [3]. Several lines of evidence suggest that environmental factors, such as viruses, diet, and gut microbes, may be associated with the initiation of islet autoimmunity and T1D progression [4,5]. However, T1D is a heterogeneous and complex disease [1]. It is thus unlikely that a single factor contributes towards autoimmunity initiation and overt disease. The identification of various endogenous and exogenous contributing factors and their interactions is essential for the early prediction and prevention of T1D.

Type 1 diabetes is preceded by the appearance of autoantibodies against β-cell antigens [6]. Increasing evidence also suggests that distinct metabolic signatures are associated with the development of T1D, already before the initiation of β-cell autoimmunity [7,8]. Recent findings suggest that children who progress to T1D have a distinct lipidomic profile already present in their cord blood, which may be helpful in the early identification of at risk children at birth [9,10]. However, more studies are needed in order to establish the link between the lipidome at birth and progression to islet autoimmunity and overt disease later in life. Here, we characterized the cord plasma lipidome in three study groups of newborn infants: (1) those who progressed to T1D (PT1D) during the follow-up, (2) those who developed at least a single islet autoantibody (Ab) during the follow-up but did not progress to T1D (P1Ab), and (3) controls (CTR) who remained autoantibody negative and healthy.

## 2. Results

We determined the umbilical cord plasma lipidome from 101 infants across three groups: PT1D (*n* = 30), P1Ab (*n* = 33), and CTR (*n* = 38), using ultra-high-performance liquid chromatography quadrupole time-of-flight mass spectrometry (UHPLC-QTOFMS). Selected clinical characteristics of the study subjects are shown in Table 1.

A total of 232 lipid species were identified and quantified using class-specific internal standards, covering the following lipid classes: cholesterol esters (CE), diacylglycerols (DG), lysophosphatidylcholines (LPC), phosphatidylcholines (PC), phosphatidylethanolamines (PE), sphingomyelins (SM), and triacylglycerols (TG).

**Figure 1:**
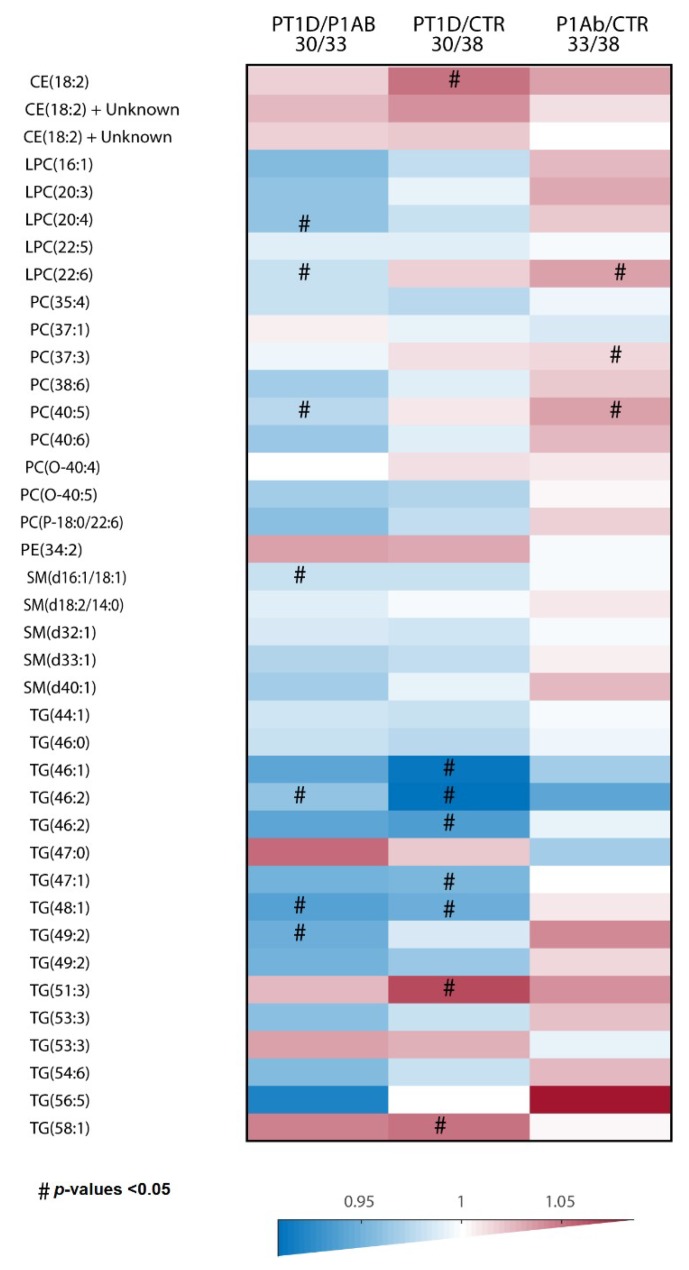
The differences in global cord plasma lipidome between the three study groups. Heat map showing 40 lipid species representative of lipid classes that change between PT1D, P1Ab and CTR. Differences in lipid concentrations were calculated by dividing the mean concentration in PT1D by the mean concentrations in P1Ab and CTR. # represents the *p*-values <0.05. Abbreviations: cholesterol ester (CE), phosphatidylcholine (PC), phosphatidylethanolamine (PE), sphingomyelin (SM), and triacylglycerol (TG), lysophosphatidylcholines (LPC).

In order to determine lipid alterations between PT1D vs. CTR, PT1D vs. P1Ab and PT1D vs. P1Ab, we performed univariate analysis, as well as multivariate analysis. The heat map in Figure 1 highlights the global lipidome that differed between the three study groups (CTR/PT1D/P1Ab). We found that seven lipids were different between PT1D and P1Ab (nominal *p*-value <0.05, Supplementary Information (S.I. 1)). These lipids included LPC (20:4), LPC (22:6), PC (40:5), SM (d16:1/18:1) or SM (d18:2/16:0), TG (46:2), TG (48:1), and TG (49:2). All of these lipids remained lower in PT1D than in the P1Ab. Similarly, we observed that eight lipids were different between CTR and PT1D (nominal *p*-value <0.05, S.I. 2). These lipids were TG (46:2), TG (46:2), TG (48:1), TG (46:1), and TG (47:1), which were downregulated among PT1D, while CE (18:2), TG (51:3), and TG (58:1) remained upregulated in PT1D compared to CTR. Furthermore, we also found four lipids being altered at a nominal *p*-value <0.05 between CTR and P1Ab (S.I. 3). All of these lipids, including LPC (22:6), PC (40:5), PC (37:3), and PC (P-18:0/22:6), were higher in the P1Ab group than in the CTR group. However, none of these lipids passed significance at the selected false discovery rate (FDR) threshold of 0.1.

**Figure 2:**
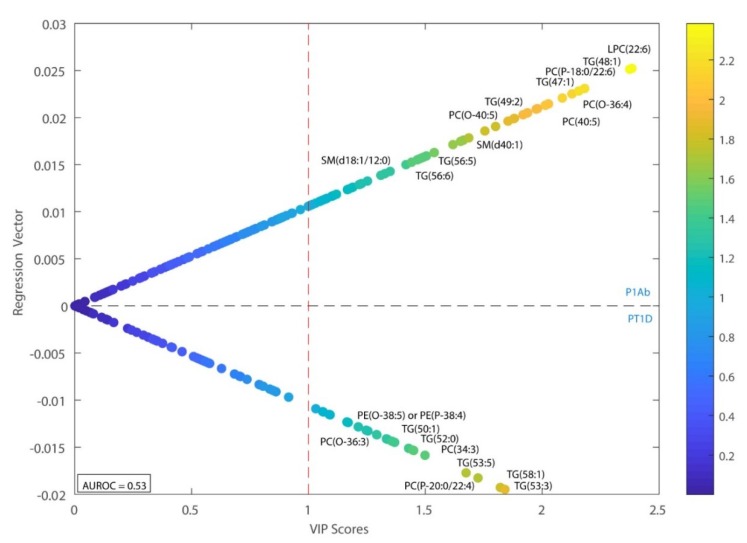
Regression coefficient plot from partial least square discriminant analysis (PLS-DA). This plot has the regression coefficient on the *y*-axis and variable importance in projection (VIP) scores on the *x*-axis. The positive and negative regression coefficients represent P1Ab and PT1D groups, respectively. Abbreviations: diacylglycerol (DG), lysophosphatidylcholine (LPC), area under the receiver operating characteristic curve (AUROC).

Multivariate models were calculated to compare case–control lipidomic patterns in relation to the progression of T1D, as well as islet autoantibody development. Principal component analysis (PCA) of the pre-processed data revealed no clear pattern or clustering of the data, and no outliers were detected. Subsequently, we applied supervised partial least square discriminant analysis (PLS-DA) models to identify differences of lipidomes between the study groups, i.e., PT1D vs. CTR, PT1D vs. P1Ab, and PT1D vs. P1Ab. Areas under the receiver operating characteristic (AUROC) and variable importance in projection (VIP) scores were used to evaluate the performance of the PLS-DA models and to identify the most discriminating lipids. PLS-DA analysis comparing PT1D and P1Ab revealed that CEs (CE (18:2)) and PEs (PE (34:2), PE (O-38:5)/PE (P-38:4), were higher in the PT1D group compared to P1Ab (VIP > 1), while most of the LPCs and SMs remained lower in PT1D than in the P1Ab group, except for LPC (18:0) and SM (d18:2/24:1) (Figure 2). Similarly, we calculated the PLS-DA models comparing PT1D vs. CTR and P1Ab vs. CTR. However, the multivariate analyses in these models revealed poor classification accuracy (AUROC approx. 0.5).

Next, we investigated whether cord plasma lipidomic signatures predicted the risk for progression to T1D and the development of islet autoantibody utilizing a logistic regression (LR) model. The lipid variables with a nominal *p*-value <0.05 in the univariate tests were used as input to build the LR models between the study cases, and a stepwise variable removal procedure was performed to find the best model for the prediction of the cases (PT1D and/or P1Ab). When comparing PT1D with CTR, the model showed good predictive accuracy, which predicted the risk for progression to T1D with AUROC = 0.84. This LR model comprised five lipid species: TG (46:2), TG (48:1), TG (46:2), CE (18:2), and TG (51:3). When applied to CTR vs. P1Ab, the LR model, which included one lipid species, PC (37:3), had less predictive accuracy (max. AUROC = 0.65).

## 3. Discussion

Our findings have provided further evidence that T1D progressors have a characteristic lipidomic profile already present at birth. Phospholipids, specifically SMs, tended to be lower in T1D progressors than in P1Ab and the CTR. Previous studies have suggested that progression to T1D is associated with decreased concentrations of major phospholipids, including SMs and PCs in cord blood [9,10]. These findings are also in line with prospective observations in children who later progressed to T1D, as well as in children with newly diagnosed T1D [7,8]. The differences observed in the present study were not as pronounced as those observed in a previous study [9]. However, in that study, the sample size allowed the PT1D group to be divided into early and late progressors (age of diagnosis below or above four years), and the distinct phospholipid signature was only identified among the early progressors.

Sphingomyelins are one of the major choline-containing phospholipids in circulation. Choline is a precursor for the biosynthesis of PCs and SMs [11,12], which are essential constituents of cellular membranes [13]. It is, however, challenging to identify the sources of the observed lipid changes in cord blood. There is evidence that cord blood lipid levels may partly reflect the maternal lipid profile during late pregnancy [14]. During pregnancy, as well as in fetal development, there is high demand of choline [15], thus it is conceivable that insufficient maternal choline availability may have mediated the downregulation in phospholipid levels in the cord blood of T1D progressors. Low choline intake can also result in low levels of triacylglycerols (TGs) [16]. Intriguingly, we also observed lower levels of several TGs species among T1D progressors, and those TGs that remained downregulated were a component of the predictive models for T1D progression.

A potential limitation of the study is that we could not investigate the association of maternal factors, such as lifestyle, diet, and body mass index (BMI), which could likely affect metabolome in both mothers and newborns. Future investigation in mother–offspring pairs will be needed to clarify the impact of the maternal factors that may lower cord blood phospholipid in relation to the progression of T1D.

The evidence suggests that SMs, as well as TGs, are potent regulators of immunogenic processes and play a potent role in inflammatory disease [17,18]. Based on our observations, we hypothesized that distinct cord blood phospholipids, as well as TGs, disturbed early immune developmental processes in T1D progressors. Further studies are clearly needed in order to elucidate the immune modulatory function of the observed lipid species during early T1D progression. Despite the current lack of mechanistic understanding of the observed lipid changes in T1D at birth, our findings suggest that lipid profiles of newborns may complement genetic testing and islet autoantibody determination for the identification of high-risk children for T1D. Clearly, further studies are needed to examine the complementary diagnostic value of these lipid signatures. The inclusion of mother–offspring pairs would also be a valuable comparison for future studies.

## 4. Materials and Methods

### 4.1. Study Design and Sampling

The study participants were selected from the prospective cohort of the Finnish Type 1 Diabetes Prevention and Prediction (DIPP) study [19]. The DIPP study has screened more than 220,000 newborn infants for HLA-conferred susceptibility to T1D in three university hospitals: Turku, Tampere, and Oulu in Finland. The subjects involved in the current study were chosen from the subset of DIPP children from the Tampere study center, as described previously [7]. Here, we analyzed 101 cord plasma samples from three study groups: (1) 30 PT1D cases, (2) 33 P1Ab cases, and (3) 38 CTR, who were observed until the age of 15 years. These study groups were matched by gender, time of birth and HLA-associated diabetes risk. Our study protocol followed the guidelines of the Declaration of Helsinki. Parents provided written consent for participation in the study. The study protocols were overseen by the ethics and research committees of the participating university hospitals. Cord blood was collected during delivery, and plasma was isolated within 30 min of collection by centrifugation at 1600 *g* for 20 min at room temperature, then aliquoted, and stored at −80 °C until analyzed.

### 4.2. Islet-Cell Autoimmunity Determination

For each study participant, we monitored the appearance of T1D-associated autoantibodies: islet cell antibodies (ICA), insulin autoantibodies (IAA), islet antigen 2 autoantibodies (IA-2A), and glutamic acid decarboxylase autoantibodies (GADA). We measured these autoantibodies at the Research Laboratory, Department of Pediatrics, University of Oulu, from the plasma samples taken during follow-up visits [6]. The ICA were detected with the use of indirect immunofluorescence, whereas the other three autoantibodies were quantified with the use of specific radio binding assays [20]. We used the cut-off limit for positivity of 2.5 Juvenile Diabetes Foundation (JDF) units for ICA, 3.48 relative units (RU) for IAA, 5.36 RU for GADA, and 0.43 RU for IA-2A. The disease sensitivity and specificity of the assay for ICA were 100% and 98%, respectively, in the fourth round of the international workshops on standardization of the ICA assay. According to the Diabetes Autoantibody Standardization Program (DASP) and the Islet Autoantibody Standardization Program (IASP) workshop results in 2010–2015, disease sensitivities for the IAA, GADA and IA-2A radio binding assays were 36–62%, 64–88% and 62–72%, respectively. The corresponding disease specificities were 94–98%, 94–99% and 93–100%, respectively.

### 4.3. Sample Preparation and Ultra High Performance Liquid Chromatography-Mass Spectrometer Analysis

The established lipidomic protocol was applied similarly, as previously described [7].

Plasma lipid extracts were prepared using a modified version of the Folch procedure: 10 µL of 0.9% NaCl, 40 µL of CHCl_3_: MeOH (2:1, *v*/*v*) [21]. For quality control, internal standards solution PE (17:0/17:0), SM (d18:1/17:0), Cer (d18:1/17:0), LPC (17:0), TG (16:0/16:0/16:0) -13C3 and PC (16:0/d30/18:1)) were added to each plasma sample, as previously described [7]. Then, the samples were vortex mixed and incubated on ice for 30 min, and centrifuged at 9400× *g* for 3 min at 4 °C. From the lower layer of each sample, we transferred 60 µL of extract to a glass vial with an insert and 60 µL of CHCl_3_: MeOH (2:1, *v*/*v*) was added to each sample. We randomized these samples and stored them at −80 °C until further analysis.

The separations were performed on an ACQUITY UPLC^®^ BEH C18 column (Waters, Milford, MA, USA, 2.1 mm × 100 mm, particle size 1.7 µm). A mass spectrometer 6550 iFunnel QTOF-MS from Agilent Technologies (Santa Clara, CA, USA), interfaced with a dual jet stream electrospray (dual ESI) ion source, was used in the study. The referenced mass solution included ions at *m*/*z* 121.0509 and 922.0098 and was introduced to the mass spectrometer through the other nebulizer in the dual electrospray ionization (ESI) ion source, using a separate Agilent series 1290 isocratic pump at a constant flow rate of 4 mL min^−1^ (split to 1:100 before the nebulizer). The acquisition mass range was *m*/*z* 100–1700 and the instrument was run in extended dynamic range mode with an approximate resolution of 30,000 full width at half maximum (FWHM), measured at *m*/*z* 1521.9715 (which is included in the tune mixture) during calibration of the instrument. MassHunter B.06.01 software (Agilent) was used for data acquisition.

### 4.4. Data Pre-Processing

MS data pre-processing was performed using MZmine 2.18.2 [22], as previously described [7]. First, crop filtering with a *m*/*z* range of 350–1700 *m*/*z* and a retention time (RT) range of 2.5 to 21.0 min was performed, then mass detection was done (noise level of 750). Second, after the chromatogram builder was set up, chromatogram deconvolution was performed with a 70% chromatographic threshold, 0.05 min minimum RT range, 5% minimum relative height, 2250 minimum absolute height, a minimum ration of peak top/edge of 1, and a peak duration range of 0.08 to 5.0. Then, isotopic peak grouping was performed with a *m*/*z* tolerance of 5.0 ppm, RT tolerance of 0.05 min, a maximum charge of 2, with the most intense isotope set as the representative isotope. Peak filter with minimum 12 data points, a FWHM between 0.0 and 0.2, tailing factor between 0.45 and 2.22 and asymmetry factor between 0.40 and 2.50, and peak list row filter, keeping only peak with a minimum of 1 peak in a row, were used. The join aligner had a *m*/*z* tolerance of 0.006, or 10.0 ppm, and a weight of 2, a RT tolerance of 0.1 min and a weight of 1, with no requirement of charge state or identity No comparison of isotope pattern was used with peak list row filter with a minimum of 10% of the samples, duplicate peak filter with a *m*/*z* tolerance of 0.006 *m*/*z* or 10.0 ppm and a RT tolerance of 0.1 min. Then, gap filling was performed using the same RT and *m*/*z* range gap filler algorithm, with an *m*/*z* tolerance of 0.006 *m*/*z*, or 10.0 ppm. Again, peak filter used the same parameters as step 6, (13) peak list row filter with a minimum of 50% of the samples was performed, enabling detection of 1084 features. Next, identification of lipids was done using a custom database search (232 identified out of 1084 features), with an *m*/*z* tolerance of 0.006 *m*/*z*, or 10.0 ppm, and a RT tolerance of 0.1 min. Normalization was performed using lipid-class-specific internal standards, using in-house developed R-script. Finally, we performed data imputation of missing values with the half of the minimum value for each lipid.

### 4.5. Statistical Analysis

All statistical analyses were performed on log2-transformed intensity data. The transformed data was mean centered and auto scaled prior to multivariate analysis. The multivariate analysis (PCA and PLS-DA) was done using the PLS Toolbox 8.2.1 (Eigenvector Research Inc., Manson, WA, USA) in MATLAB 2017b (Mathworks, Inc., Natick, MA, USA). The area under the receiver operating characteristic was calculated to evaluate the ability of the discrimination model to correctly classify the samples. All PLS-DA models were cross validated by splitting the dataset into test sample subsets for each sub-validation experiment, using a random subset with ten data splits and twenty repeats. Logistic regression (LR) analyses were computed in MATLAB 2017b using the statistical toolbox and PLS toolbox (v8.1, Eigenvector labs, Manson, WA, USA). For the LR classification model, 1000 cross-validated models were generated from the lipid variables, which had nominal *p*-value <0.05 in the univariate tests. Each model contained 70% of the original data, with 30% used as a testing set. The performance of the models was evaluated based on the AUC of the resultant receiver operator curve (ROC). To select the optimal combination of metabolites for the linear regression, we introduced metabolites in an iterative manner, starting with all metabolites individually who met the *p*-value cut off. The highest AUC value model was then identified. This metabolite was then used in combination with all remaining metabolites. New models were generated in this iterative fashion until the AUC value started to fall. The optimal combination of variables was then determined based on the maximum AUC value.

For univariate analysis, Wilcoxon rank–sum test was performed for comparing the two study groups of samples (e.g., PT1D vs. P1Ab). The resulting *p*-values <0.05 were considered significantly different among the group of hypotheses tested in a specific age cohort. The resulting nominal *p*-values were corrected for multiple comparisons, using the Benjamin and Hochberg approach [23]. All of the univariate statistical analyses were computed in MATLAB 2017b using the statistical toolbox. The fold difference was calculated by dividing the mean concentration of a lipid species in one group by another, for instance, mean concentration in the PT1D by the mean concentration in P1Ab, and then illustrated by heat maps.

## Figures and Tables

**Table 1:** Anthropometric characteristics of the study population.

	PT1D (*n* = 30)	P1Ab (*n* = 33)	CTR (*n* = 38)
**Gender (girls, boys)**	(12, 18)	(12, 21)	(14, 24)
**Age at time of diagnosis (years)** **(mean ± SE)**	4.94 ± 0.56	NA	NA
**Age at time of first seroconversion (years)** **(mean ± SE)**	1.28 ± 0.11	3.03 ± 0.45	NA

Abbreviations: Children who progressed to T1D (PT1D), who developed at least a single antibody but did not develop T1D during the follow-up (P1Ab), and control (CTR) subjects who remained islet autoantibody negative during the follow-up. Standard error (SE); Not applicable NA.

## Supplementary Materials

The following are available online at http://www.mdpi.com/2218-273X/9/1/33/s1, Table S.I. 1: Differential plasma lipids between P1Ab and PT1D, S.I. 2: Differential plasma lipids between CTR and PT1D, S.I. 3: Differential plasma lipids between CTR and P1Ab.
